# Characterization of Fluorescence Tracers for Thermometry and Film Thickness Measurements in Liquid Coolants Relevant for Thermal Management of Electric and Electronic Components

**DOI:** 10.3390/s22228892

**Published:** 2022-11-17

**Authors:** Matthias Koegl, Moritz Delwig, Lars Zigan

**Affiliations:** 1 Institut für Thermodynamik, Professur für Energiewandlung, Fakultät für Luft- und Raumfahrttechnik, Universität der Bundeswehr München (UniBw M), D-85577 Neubiberg, Germany; 2 Erlangen Graduate School in Advanced Optical Technologies (SAOT), Friedrich-Alexander-Universität Erlangen-Nürnberg (FAU), D-91052 Erlangen, Germany

**Keywords:** two-color LIF technique, liquid temperature, heat transfer fluids, cooling

## Abstract

This study investigated a novel two-color LIF (laser-induced fluorescence) technique for thermometry in coolants relevant for electric components. In principle, this diagnostic enables thermometry in liquid flows but also a simultaneous determination of film thickness and film temperature, which is relevant, e.g., for jet impingement cooled electric components. Temperature measurements are based on a temperature-sensitive intensity ratio of special tracers realized by suitable band pass filters within the respective emission spectra. For this purpose, the heat transfer fluids Fragoltherm F12, Marlotherm LH, and a water–glycol mixture WG20 (80 vol.% water, 20 vol.% glycol) and its individual components were doped with suitable tracers. The tracer Eosin-Y was utilized for polar coolants (water, WG20, and glycol) and Nile red was utilized for non-polar solvents (Fragoltherm F12 and Marlotherm LH). The spectral LIF intensities were recorded for a wide range of temperatures (253–393 K), which are relevant for cooling of electric motors, batteries, and power electronics. Furthermore, absorption spectra were analyzed as well. The temperature-dependent fluorescence measurements revealed different behavior for the polar and non-polar solvents. A temperature increase in the polar solvents (water, WG20, glycol) led to a spectral shift of the emission peaks of Eosin-Y towards longer wavelengths (red-shifted), while the peaks of Nile red in the non-polar solvents (Fragoltherm F12 and Marlotherm LH) showed an opposite behavior and were blue-shifted. The highest average temperature sensitivity was achieved for Marlotherm LH (4.22%/K), followed by glycol (1.99%/K), WG20 (1.80%/K), water (1.62%/K), and Fragoltherm F12 (1.12%/K). These sensitivities are similar to or even much higher than the literature data of other LIF tracers, which were, however, not determined in those coolants. Consequently, the two novel proposed dyes for the studied heat transfer liquids enable a reliable temperature determination.

## 1. Introduction

Electric and electronic devices such as motors, generators, power controllers, and battery systems, among others, require complicated thermal management solutions since high heat flux dissipation rates and homogeneous temperature distributions are indispensable. Large heat flux dissipation rates in regards to electric applications are often realized by immersion cooling [1,2], heat pipes [3,4], or impingement cooling [5,6,7,8,9,10]. Immersion cooling, where the components are immersed in a cooling fluid, is not the most weight-efficient way of cooling and mainly suitable for stationary devices such as transformers, generators, motors, battery and server applications (electric, electronic, or IT components), and, e.g., steel treatment (steel industry). The principle of local heat transfer using heat pipes is based on phase transition to transfer heat between two solid interfaces. Heat pipes find their application in very compact units (e.g., laptops, CPUs, etc.), where limited space is available and a demand for high heat flux dissipation is required [3,4], especially for the minimization of hot spots. Jet or spray impingement cooling is based on jet/small atomized droplets impacting the hot surface, forming a liquid film and leading to high heat dissipation flux. Here, sprays enable an economic liquid consumption combined with high efficiency and are described for this reason in more detail [5]. Commonly monodisperse (uniform size of droplets) [9,10] and polydisperse sprays [6,7,8,11] are distinguished. A lot of nozzles were developed over the last decades to create various spray geometries (e.g., full cone, hollow cone, flat fan, air–mist nozzle) [5]. An optimization of the spray impingement process requires a deep understanding of the individual sub-processes. In addition to the atomization process, the film temperature and film thickness determine the heat flux and the required heat transfer fluid volume flow.

The subsequent paragraphs are structured as follows. First, an overview of well-established optical spray diagnostic techniques for droplet size and temperature distribution based on laser-induced fluorescence (LIF) is provided. These aspects and LIF dyes are also relevant for the present study. Second, possible available film thickness measurement techniques are summarized. Finally, open tasks for the further development of the LIF technique for combined thermometry and film thickness measurements define the scope of the present paper.

The liquid spray structure can be characterized by various non-invasive techniques, which were developed and improved over the last decades. A 2D determination of droplet size and temperature can be achieved by LIF [12]. LIF/Mie droplet sizing, also known as *d*_32_ droplet sizing, enables the planar determination of the droplet size dispersion in terms of Sauter mean diameter (SMD, *d*_32_) within a spray [13,14,15,16,17,18,19,20,21]. Here, Mie scattering was combined with LIF. The LIF/Mie technique is based on the d^3^ dependence of the LIF signal and the d^2^ dependence of the Mie signal [13,14,15,16,17,18,22,23,24]. A quantitative characterization of the spray in terms of absolute SMD is only possible with adequate calibration such as phase doppler interferometry (PDI) or micrometric droplet measurements with a droplet generator [14,25,26]. In addition to the LIF/Mie approach, other techniques such as the Raman/Mie ratio [27], the polarization ratio [28,29,30,31,32], and MDR (morphology-dependent resonances) images of micro droplets [33] enable a determination of a 2D droplet size distribution.

The LIF signal is usually created by a tracer (or “dye”) dissolved in a liquid [21] or by the liquid itself (e.g., aromatic components in multi-component fuel) [34]. The LIF signal may be very temperature sensitive and it depends on the absorption and emission properties of the tracer, the solvent itself, and the utilized illumination source (e.g., laser, LED, and the respective excitation wavelength and irradiation).

**Figure A1:**
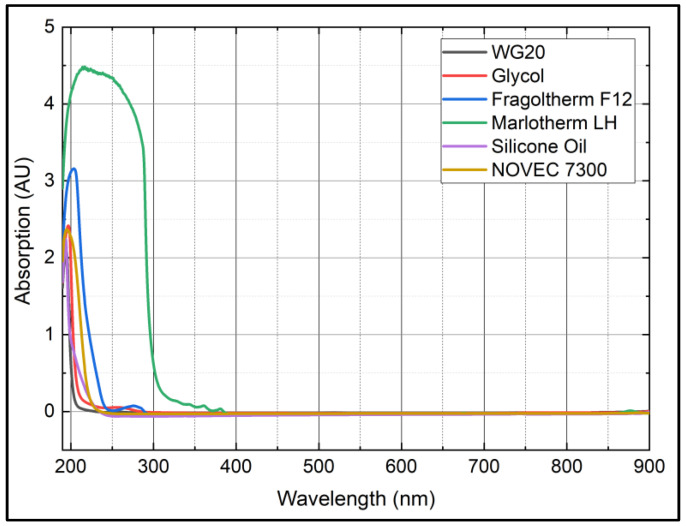
Absorption of various heat transfer fluids, 293 K.

Common tracers for studying the mixture formation and temperature in the gas phase are acetone, 3-pentanone, anisole, toluene, 1-methylnaphthalene, and triethylamine [35,36,37,38]. However, in the liquid phase, these tracers show only a low temperature sensitivity so that they are probably not applicable for thermometry (see, e.g., Geiler et al.) [39]. Furthermore, these tracers are excited in the UV range, which may overlap with the absorption spectrum of the solvents (e.g., of heat transfer oils; see Appendix A, Figure A1).

Suitable dyes for fluorescence studies in liquids are, e.g., rhodamine B, fluorescein, pyrromethene, and coumarin [12,40,41,42]. All of them show a distinct temperature sensitivity in some solvents, which may be utilized for two-color LIF thermometry. This ratio-based technique exploits the shift and broadening of the fluorescence spectra with temperature. An appropriate selection of two detection channels (commonly realized by suitable band pass filters) enables a determination of the temperature after careful calibration. Although coumarin shows a superior temperature sensitivity, it must be excited in the UV wavelength range, which may be disadvantageous for certain solvents that show absorption in the UV spectrum as well. Rhodamine B works well in ethanol and water [43,44,45,46,47]. Fluorescein is often used in combination with water and ethanol [48,49,50]. Pyrromethene is mainly used in alkanes (dodecane), ketones (3-pentanone), and alcohols [45,51,52,53,54,55,56]. Coumarin is mainly used for two-color LIF thermometry in ethanol [42]. Nile red was proposed as a dye for thermometry in ethanol/iso-octane mixtures; it can be utilized for the determination of the composition of butanol/decane mixtures [57,58]. Nile red is less temperature sensitive in fuels such as E20 (80 vol.% iso-octane, 20 vol.% ethanol) and E40, while it shows a high temperature sensitivity for kerosene and its biofuel blends [59].

A lot of non-invasive techniques for the characterization of the liquid film structure have been developed within the last decades. The wall heat flux, which determines the minimal packing density of high-power applications, is commonly characterized by wall-inserted thermocouples [8,11,60]. The heat flux can also be estimated with, e.g., infrared thermography (IRT) [9,10,61,62]. The Weber number and the mass flux are two common parameters to classify and quantify the spray cooling of hot surfaces [5]. The liquid film thickness can be determined by various techniques. Laser-induced fluorescence is mainly used for thick films (10 µm–100 µm) [63,64,65,66,67,68,69], laser absorption techniques such as laser light absorption for large films (up to 5 mm) [70] as well as laser absorption spectroscopy (up to 1600 µm) [71,72,73]. Refractive-index matched (RIM) imaging is used for very thin films (0.1–3µm), being relevant, e.g., for fuel film formation and evaporation in IC engine environments (such as direct-injection spark-ignited engines) [74,75].

Until now, only a few investigations studied a simultaneous detection of film thickness and temperature. Schagen et al. investigated the film thickness and temperature in a laminar, wavy liquid film of water doped with biacetyl (2,3-butanedione). The developed techniques use the fact that biacetyl emits phosphorescence as well as fluorescence when illuminated with UV light [76]. The temperature profile in the liquid film is determined by the phosphorescence; the local film thickness is determined by the fluorescence. Borgetto et al. investigated the film thickness and temperature of a liquid heptane film along a wall with a low-coherence interferometry technique. The technique showed some limitations, e.g., regarding minimum thickness measurement, which is fixed by the coherence length of the light source (20 µm @ 1310 nm) and the influence of surface waves on the measurement results [77]. Huang et al. investigated the thickness and temperature profile of a lubricant film during a machining process based on fluorescence. The fluorescence tracer “fluorescence pink” was used as a temperature-insensitive probe and europium 3 thenoyltrifluoroacetonate (EuTTA) was used as a temperature-sensitive probe [69]. Wu et al. determined the film thickness and temperature of water on a metal plate with a diode laser absorption spectroscopy method. Their results are in good agreement with thermocouple (temperature deviation: 2.0%) and ultrasonic pulse-echo method (film thickness deviation: 3.3%) measurements [73]. Collignon et al. investigated the characteristics of thin liquid films flowing down a heated and inclined plane based on a two-color LIF technique. They measured the averaged film temperature and film thickness of water simultaneously and quantified the heat transfer coefficient [78].

The present study focused on the development of a two-color LIF thermometry technique for various coolant liquids such as heat transfer oils and water/glycol mixtures. The goal was to use one tracer in combination with two spectral detection channels to determine the liquid temperature in various cooling applications. The technique should also enable the measurement of film thickness and temperature simultaneously in spray cooling experiments. The heat transfer liquids were selected in view of electrical cooling applications (e.g., motor, generator, battery). For this purpose, a spectral investigation of commonly used heat transfer fluids either doped with a suitable tracer (Eosin-Y, Nile red) in terms of absorption and emission measurements was derived. First, the tracer concentration-dependent absorption and emission measurements were carried out. Second, the effect of photo-dissociation was studied. Third, the temperature-dependent emission spectra were investigated, and suitable filters were suggested and validated. Temperature sensitivities were determined for the respective dye/cooling fluid combination. Finally, a brief conclusion and an outlook of the desired two-color LIF technique was provided.

## 2. Description of the Experiment

### 2.1. Fluorescence Spectroscopy Setup

**Figure 1:**
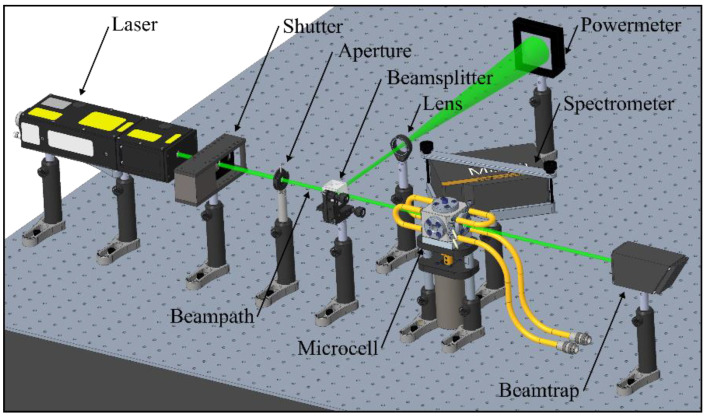
Optical setup for the fluorescence measurements.

The experimental setup is shown in Figure 1. The probe volume was illuminated with a pulsed Nd:YAG laser (model Ultra50, bigSky, Mountain Village, MT, USA; wavelength 532 nm, repetition rate 10 Hz, pulse width < 8 ns). A remote-controlled external shutter enabled probe illumination only during measurements, keeping possible photo-dissociation effects as low as possible. A downstream aperture cut the initial laser beam cross section down to 2.8 mm. The beam was divided by a 50/50 beam splitter to ensure simultaneous monitoring of the laser fluence (power meter: model QE50LP-S-MB-INT-D0, Gentec Electro-Optics, Quebec, QC, Canada) during the measurements. The transmitted beam passed the measurement volume in the cell (for temperature-dependent measurements) or a cuvette (for concentration studies), respectively. A spectrometer (model: Maya 2000-Pro, Ocean Optics, USA, wavelength range 200.5–1120.4 nm, pixels 2048, slit size 25 µm, integration time 100 ms; 50 subsequent spectra were averaged for each measurement) recorded the LIF spectra under a detection angle of 90°. A cuvette (model: 101-10-40, Hellma Analytics, edge length 10 mm, 3.5 µL) was used for the concentration-dependent measurements, while the temperature-dependent measurements were conducted in a specially designed micro cell. The micro cell featured four optically accessible windows (“½” sapphire windows, optical access diameter: 9 mm, inner distance between two windows: 19.1 mm). A magnetic stirrer (stir bar: 8 mm × 3 mm, 1500 rpm) ensured a homogenous temperature distribution of the probe volume, which was monitored by two thermocouples (type K, tc-direct GmbH, Kitzingen, Germany). The built-in cooling/heating circuits driven by a recirculation thermostat (model: C50P, Thermo Fisher Scientific, Waltham, MA, USA) enabled a wide range of investigated temperatures. A connected pressurized tank (0.8 MPa) enabled refilling the preheated/precooled cell in the case of photo bleaching sensitive liquid tracer mixtures. Further details of the measurement cell used are described in [57,59,79].

### 2.2. Absorption Spectroscopy Setup

The absorption measurements were carried out with a UV/VIS spectrometer (model V-750, JASCO, Tokyo, Japan, light sources: halogen and deuterium lamps, wavelength range 190 nm–900 nm, 3551 pixels, spectral bandwidth 2 nm, scan speed 200 nm/min). The cuvette specified in the fluorescence measurement setup (Section 2.1) was used for the absorption measurements as well, since only spectra at room temperature (293 K) were recorded.

## 3. Coolants and Tracers Used

In the present study, the emission and absorption signals of commercially available heat transfer fluids doped with suitable fluorescence tracers were investigated. For this purpose, we investigated the heat transfer fluids Fragoltherm F12 (Fragol AG, Mülheim, Germany), Marlotherm LH (Sasol Germany GmbH, Hamburg, Germany), and a water–glycol mixture WG20 (80 vol.% water, 20 vol.% glycol) and its individual components. Silicone oil (Type: M40.165/220.10, Peter Huber Kaeltemaschinenbau AG, Offenburg, Germany) and the engineering fluid NOVEC 7300 (3M, St. Paul, MI, USA) showed no sufficient solubility in combination with the tracer Nile red since the tracer was only partially soluble in the fluids and floating particles were present. The same was true for the dye Eosin-Y. Consequently, NOVEC 7300 and silicone oil could not be analyzed in the framework of this study. The chemical and physical properties of the investigated liquids are shown in Table 1.

Nile red (C_20_H_18_N_2_O_2_, Sigma Aldrich: Bellefonte, PA, USA,) is a well-known fluorophore; the applications of Nile red were initially based in microfluidic systems and biology [85,86,87]. Recent studies utilized the tracer for planar droplet sizing of fuel sprays [57,59,88]. Its aromatic ring structure features polar substituents. The polar substituents lead to a high sensitivity to the chemical and physical environment of surrounding solvent molecules [89]. Nile red is soluble in alkanes and real-world fuels [90]. A minimum of 3.75 mg of Nile red was weighed with a high-precision analytical scale (Mettler Toledo XS 205, proofed repeatability 0.05 mg). The tracer was completely dissolved in the investigated oil–tracer mixtures. Nile red has a melting point of 476–479 K) [91], but this temperature is above the maximum tested temperature in the present micro cell setup.

Eosin-Y (C_20_H_6_Br_4_Na_2_O_5_, Sigma Aldrich; here a solution of 5 wt% in H_2_O was utilized) is a solid acid xanthene (natural ionic) tracer; in addition to uses in medicine [92], biology [93], and as groundwater migration tracer [94], it is also used for planar droplet sizing for alcohol and water sprays [19,20,21,95]. Eosin-Y is not soluble in alkanes and, e.g., gasoline [90]. The liquid Eosin-Y solution was pipetted with a microliter pipette (Reference 2G (10–100 µL), Eppendorf AG, Hamburg, Germany). Eosin-Y has a melting point of 528–543 K [96], but this temperature is above the maximum tested temperature in the present micro cell setup.

For the present study, tracer concentrations of 0.29–37.5 mg/L were investigated. The various investigated tracer concentrations were generated by diluting the initial liquid–tracer mixtures.

## 4. Results

The following results section is structured as follows. First, the effect of the tracer concentration on the absorption and fluorescence spectra (excitation wavelength: 532 nm) of the liquid–tracer mixtures is shown. Second, the effect of photo-dissociation is demonstrated. Third, the temperature influence on the emission spectra is analyzed. Finally, a brief discussion of the data is presented. All spectral results are presented in the visible wavelength region (380–780 nm), which is most relevant for the absorption and emission of Eosin-Y and Nile red.

### 4.1. Concentration-Dependent Measurements

#### 4.1.1. Absorption

**Figure 2:**
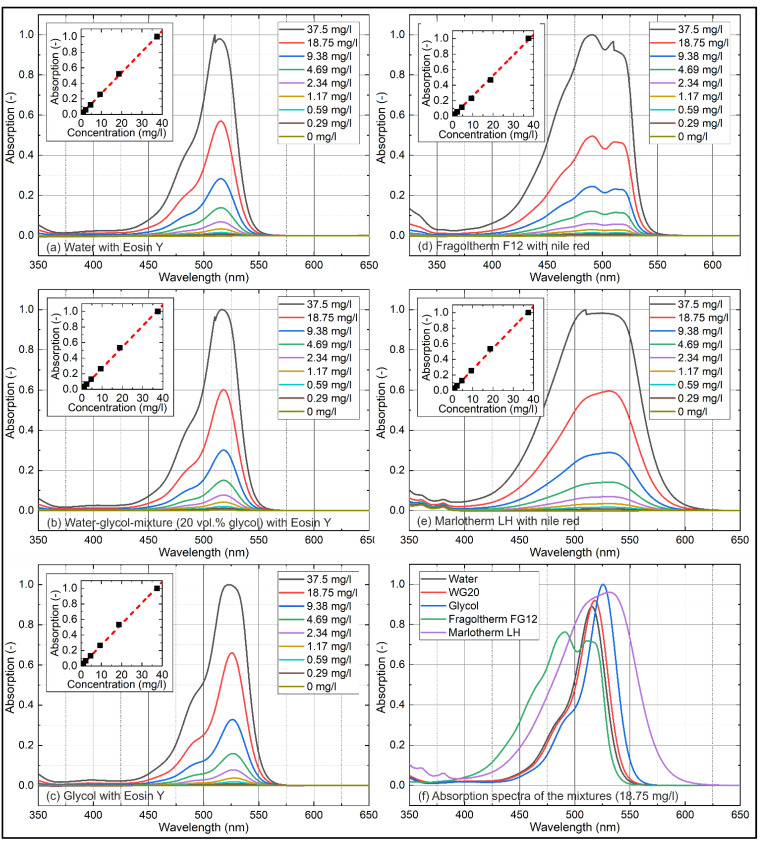
Concentration-dependent absorption spectra of Eosin-Y (**a**–**c**) and Nile red (**d**,**e**) in various solvents; comparison of absorption spectra of Nile red and Eosin-Y in various liquids at a fixed tracer concentration of 18.75 mg/L (**f**), 293 K.

The concentration-dependent absorption measurements for various solvents are shown in Figure 2. Results are normalized to the highest absorption. A comparison of all investigated mixtures normalized to the highest absorbing solvent at a tracer concentration of 18.75 mg/L is presented in Figure 2f. Eosin-Y mixed solvents (a,b,c) showed a broad absorption between 450 nm and 575 nm and were characterized by a single peak (water: 515.5 nm; WG20: 518 nm; glycol: 525.5 nm). The Nile red mixtures (d,e) showed an even broader absorption between 375 nm and 575 nm (Fragoltherm F12) and 400 nm and 625 nm (Marlotherm LH). The absorption signal of Marlotherm LH was characterized by a single broad absorption peak at 532 nm, while the absorption signal of Fragoltherm F12 showed a double peak (first peak (more pronounced): 491 nm; second peak (less pronounced): 512 nm) similar to Jet-A1 (pure and blended with HEFA (30 vol.% and 50 vol.%) or farnesane (10 vol.%)) investigated in an earlier study [59]. The absorption measurements (inserted diagrams (integrated individual spectra between 400 nm and 600 nm) in Figure 2) showed a linear behavior for all investigated solvents for all concentrations. The coefficients of determination R^2^ for the linear fitting curves displayed in Figure 2 are presented in Table 2 and confirmed the linearity of the concentration-dependent absorption measurements for all investigated tracer concentrations.

A comparison of the absorption signals at a fixed tracer concentration of 18.75 mg/L Eosin-Y/Nile red (Figure 2f) revealed the highest peak absorption for glycol followed by Marlotherm LH, WG20, water, and Fragoltherm F12.

#### 4.1.2. Emission

**Figure 3:**
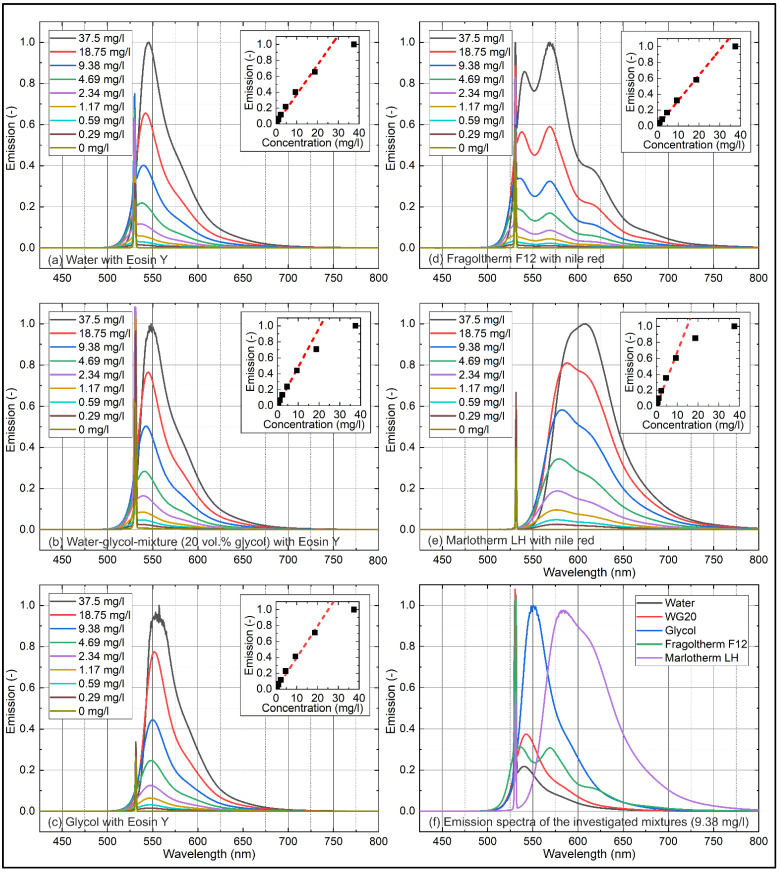
Concentration-dependent emission spectra of Eosin-Y (**a**–**c**) and Nile red (**d**,**e**) in various solvents; comparison of various liquids at a fixed tracer concentration of 9.38 mg/L (**f**), 293 K.

**Figure A2:**
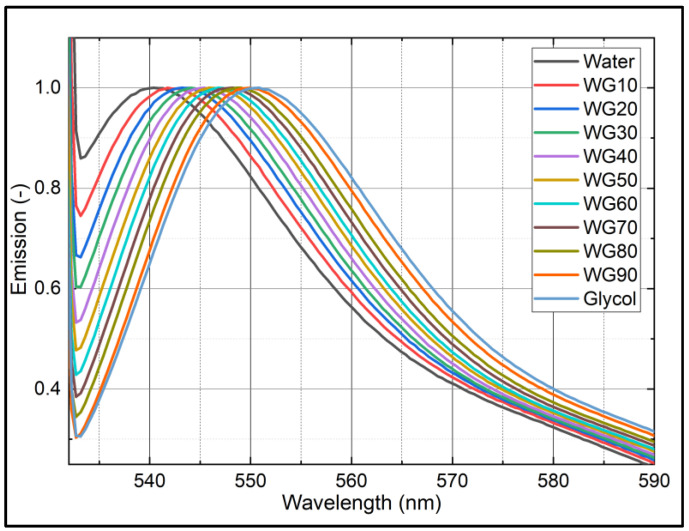
Normalized emission spectra of Eosin-Y (9.38 mg/L) in various water glycol mixtures, 293 K.

The concentration-dependent emission measurements for various solvents are provided in Figure 3. Results are normalized to the highest emission; a comparison of all investigated mixtures normalized to the highest emission at a tracer concentration of 9.38 mg/L (linearity limit of emission measurements) is given in Figure 3f. The Eosin-Y mixed solvents (a,b,c) showed a broad emission between 500 nm and 700 nm and were characterized by a single peak (water: 540.5 nm; WG20: 542.5 nm; glycol: 550.5 nm; all @37.5 mg/L). The peaks were shifted with decreasing tracer concentration (9.38 mg/L → 0.26 mg/L) towards shorter wavelengths. An increasing glycol content led to a decrease in the concentration-dependent spectral shift (water: 5 nm; WG20: 4 nm; glycol: 3 nm). Further fluorescence measurements presented in the Appendix A (see Figure A2) revealed a linear shift of the maxima with increasing glycol concentration (@9.38 mg/L) towards longer wavelengths. The Nile red mixtures (d,e) showed a broader emission between 500 nm and 750 nm (Fragoltherm F12) and 525 nm and 800 nm (Marlotherm LH). The emission signal of Marlotherm LH was characterized by a single broad emission peak at 584 nm (@37.5 mg/L), while the emission signal of Fragoltherm F12 showed a double peak (first peak: 536.5 nm; second peak: 569.0 nm; both @37.5 mg/L) similar to Jet-A1 and its biofuel blends [59].

While the absorption investigations revealed a more pronounced first peak for all concentrations, the emission results showed a different behavior. At higher tracer concentrations (>9.38 mg/L), the second peak dominated the first peak; at lower concentrations (≤9.38 mg/L), the first peak dominated the second peak. Marlotherm LH showed a similar behavior as the polar solvent tracer mixtures with a 6 nm shift (9.38 mg/L → 0.26 mg/L) of the peak with decreasing dye concentration towards shorter wavelengths, while Fragoltherm showed no tracer concentration-dependent spectral shift at all. The emission measurements (inserted diagrams (integrated individual spectra between 536 nm and 800 nm to exclude laser peak) in Figure 3) showed a linear behavior for all investigated solvents for concentrations up to 9.38 mg/L. Beyond this concentration, the distinct absorption of transmitted laser light along the beam path took place and led to a decrease in the increasing fluorescence intensity. The coefficients of determination R^2^ for the linear fitting curves (up to 9.38 mg/L) displayed in Figure 2 are shown in Table 3 and confirmed the linearity of the concentration-dependent emission signals.

A comparison of the emission signals at a fixed tracer concentration of 9.38 mg/L Eosin-Y/Nile red (Figure 3f) revealed the highest emission for glycol followed by Marlotherm LH, WG20, water, and Fragoltherm F12. These results were in agreement with the absorption measurements at 532 nm (see Figure 2f).

### 4.2. Photo-Dissociation

**Figure 4:**
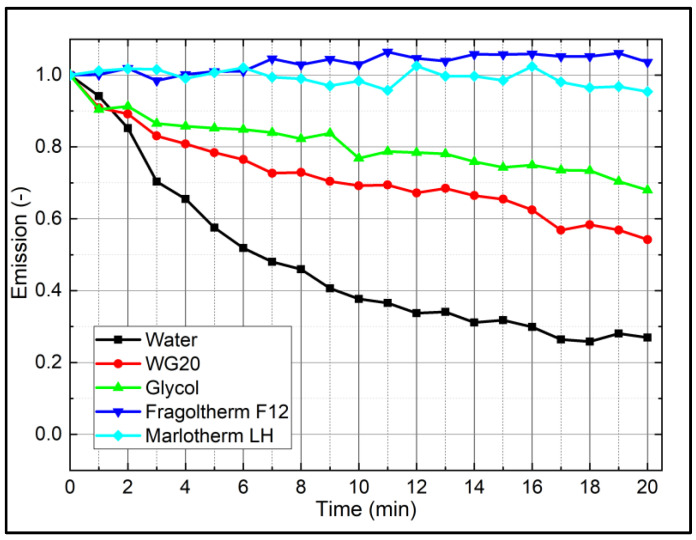
Photo-bleaching effect of the investigated liquid–tracer mixtures (9.38 mg/L), 293 K.

Photo-dissociation is an effect, which leads to a reduction in the LIF signal with time under continuous illumination (e.g., at constant laser fluence). The investigations were carried out as follows. The liquid–tracer mixtures were constantly illuminated for 20 min. The LIF spectra were recorded every 60 s, and the individual spectral fluorescence intensities (interval between 536 nm and 800 nm, to exclude the laser peak on the individual spectrum) were summed up (see Figure 4). The measurements revealed that Fragoltherm F12 and Marlotherm LH showed no significant change of the LIF signal with time, while Eosin-Y solvents showed a non-neglectable decay of the LIF signal with increasing illumination time. Here, water showed the highest intensity decrease (decay: −73%) followed by WG20 (decay: −46%) and glycol (decay: −32%). For the design of the test rigs, these investigations had to be taken into account. The photo-dissociation was especially taken into account for spectral measurements within a cuvette, closed cooling system, or at spray cooling measurements with circulation (and reuse) of the liquid–tracer mixtures. Spray investigations in spray chambers, where the fuel spray was only illuminated once and the solvent/tracer mixture was not reused, were not affected by photo-dissociation.

### 4.3. Temperature-Dependent Emission Spectra

**Figure 5:**
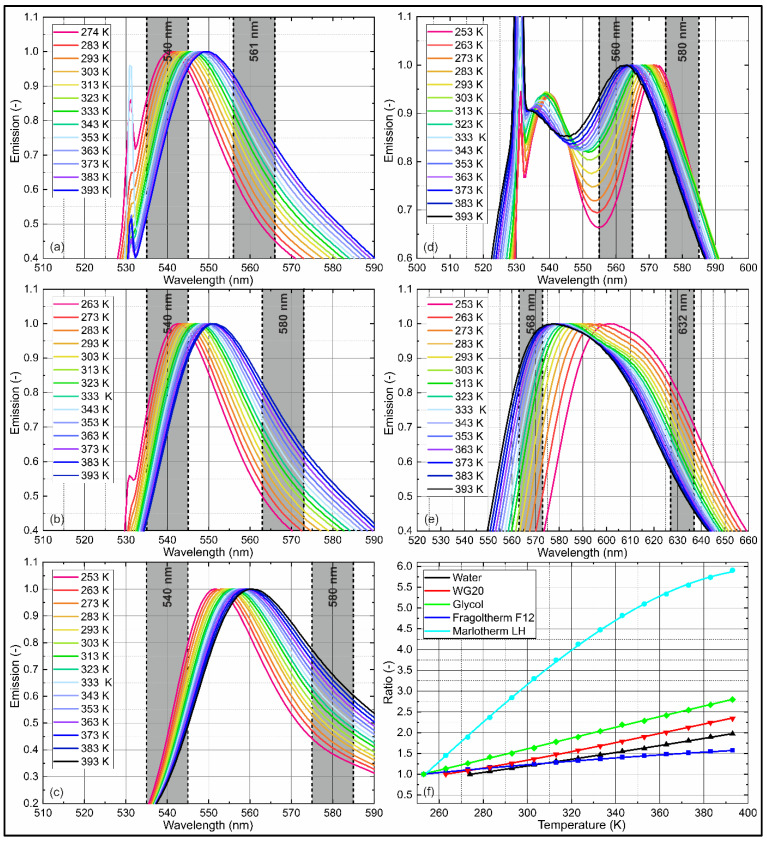
Normalized emission spectra of Eosin-Y/Nile red (9.38 mg/L) in various solvents ((**a**): water, (**b**): WG20, (**c**): glycol, (**d**): Fragoltherm F12, (**e**): Marlotherm LH) at various temperatures with suitable filters for film temperature measurements and corresponding temperature-sensitive intensity ratios (**f**).

Film temperature measurements are based on a temperature-sensitive intensity ratio. The intensity ratio is usually realized by suitable band pass filters within the respective emission spectra. The suggested band pass filters (Edmund Optics, diameter: 25 mm, FWHM: 10 nm, OD4; listed in Table 4) were inserted in the respective temperature-dependent emission spectra of the investigated liquid–tracer mixtures shown in Figure 5. The filters were chosen in order to achieve the highest possible temperature sensitivity. In the planned subsequent planar measurements, two filters were installed, enabling the use of a custom camera system with image splitter, where two individual cameras (equipped with band pass filters) were connected with one objective. Here, besides the usual sCMOS cameras, the use of high-speed cameras was also achievable. A similar setup based on two sCMOS cameras was used in earlier investigations [20,25,26,88].

The temperature-dependent emission spectra of the investigated liquid–tracer mixtures, suitable filters, and the corresponding sensitivity curves are shown in Figure 5 for a wide range of temperatures.

Water, WG20, and glycol doped with Eosin-Y (Figure 5a–c) showed a similar behavior. A temperature increase led to a spectral shift of the peaks towards higher wavelengths. Here, the left flank was unchanged, while the right flank was shifted towards longer wavelengths. This behavior was suitable for the determination of the liquid temperature using a two-color detection scheme. The spectral shift decreased slightly with an increasing temperature. Since water freezes at 273 K (0.1 MPa), the lowest temperature measurement of water doped with Eosin-Y was conducted at 274 K. In the case of WG20, the first measurement point was 263 K. The spectral shift for all three mixtures within the investigated temperature intervals was approximately 9 nm (water: 540.9–549.7 nm; WG20: 542.7–551.5 nm; glycol: 551.7–560.5 nm).

The temperature-dependent emission spectra of Fragoltherm F12 and the respective band pass filters are shown in Figure 5d. A temperature increase led to a spectral shift of the two peaks towards shorter wavelengths. A temperature increase led to a decrease in the first peak relative to the dominating second peak and to an increase in the signal minimum between the two peaks. The right flank stayed unchanged, while the left flank shifted to shorter wavelengths with an increasing temperature. The temperature-dependent spectral shift was equally spaced within the investigated temperature interval. The second peak dominated the first peak within the investigated temperature interval. The full spectral shift was approximately 10 nm (562.6–572.0 nm) within the temperature range.

The temperature-dependent emission spectra of Marlotherm LH and the respective band pass filters are shown in Figure 5e. A temperature increase led to a spectral shift of the peak towards shorter wavelengths, and the right flank stayed unchanged while the left flank shifted with an increasing temperature towards shorter wavelengths, as with Fragoltherm F12. The spectral shift decreased slightly with and increasing temperature, as with water, WG20, and glycol. The full spectral shift was approximately 24 nm (577.7–601.5 nm) within the temperature range.

The intensity ratios were determined using a multiplication of the fluorescence spectra with the respective filters (rectangular filter, center wavelength ± 0.5 FWHM).

The temperature-sensitive intensity ratios and the corresponding fitting curves (parameters and R^2^; see Table 5) are shown in Figure 5f. The average temperature sensitivity of the intensity ratio was highest for Marlotherm LH (4.22%/K), followed by glycol (1.99%/K), WG20 (1.80%/K), water (1.62%/K), and Fragoltherm F12 (1.12%/K). The sensitivities were comparable to the literature data in this field (however, in different solvents) and enabled a reliable temperature determination of the liquid phase of the investigated heat transfer fluids. For example, Vetrano et al. calculated a temperature sensitivity (297 –328 K) of a flashing jet (ethanol with rhodamine B) of 0.7%/K. [46]. Mishra et al. investigated the temperature sensitivity (298–338 K) of rhodamine B in ethanol (on average, 2.93%/K) and butanol (2.89%/K) and fluorescein in ethanol (1.27%/K) and butanol (1.42%/K) in a temperature-controlled cuvette [40]. Palmer et al. measured the temperature (285–321 K) of micro droplets and calculated a temperature sensitivity of pyrromethene in ethanol of 1.2%/K [52]. Prenting et al. characterized tracers for two-color LIF thermometry (296–348 K) in sprays. They deduced a temperature sensitivity of coumarin in ethanol of 1.2%/K.

The temperature for the various liquid–tracer mixtures can be determined with the intensity ratio *r_Temperature_* (using the following polynomial equation):

(1) $$T(K)=p_{1}\cdot r_{{}_{Temperature}}^{3}+p_{2}\cdot r_{{}_{Temperature}}^{{}^{2}}+p_{3}\cdot r_{{}_{Temperature}}+p_{4}$$

The fitting parameters and the corresponding coefficient of determination are shown in Table 5. The ratio *r_Temperature_* can be determined using the ratio of the two products of the transmission curves *τ* of the respective filters and the fluorescence signal LIF (when other efficiencies of the optical setup (e.g., cameras) are neglected):

(2) $$r_{{}_{Temperature}}=\frac{\sum \tau_{Filter\_1}\cdot I_{LIF}}{\sum \tau_{Filter\_2}\cdot I_{LIF}}$$

## 5. Conclusions and Future Work

A characterization of the absorption and emission of two dyes dissolved in different coolants was conducted in order to develop a thermometry technique based on two-color LIF. Here, the tracer Eosin-Y was utilized for polar solvents (water, WG20, and glycol) and Nile red was utilized for non-polar solvents (Fragoltherm F12 and Marlotherm LH). For this purpose, temperature-dependent laser-induced fluorescence spectra (253–393 K) were recorded using a specially designed micro cell. First, the influence of the tracer concentration on the linearity of the absorption and emission spectra was studied. The investigation revealed photo-dissociation effects in the case of water, WG20, and glycol, while it was neglected for Fragoltherm F12 and Marlotherm LH.

The temperature-dependent fluorescence measurements revealed different behavior for the polar and non-polar solvents. The peak and the right flank of the emission of Eosin-Y in polar solvents were shifted to longer wavelengths with an increasing temperature while the left flanks stayed unchanged. The fluorescence of Nile red in unipolar solvents showed a shift of the peak and the left flank to shorter wavelengths while the right flank stayed unchanged. The detection of the liquid temperature was based on intensity ratios realized by band pass filters. The suggested filters enabled a temperature-sensitive intensity ratio with the largest sensitivity for Nile red in Marlotherm LH (4.22%/K) followed by Eosin-Y in glycol (1.99%/K), WG20 (1.80%/K), water (1.62%/K), and Nile red in Fragoltherm F12 (1.12%/K). These sensitivities are similar or even much higher than the literature data of other LIF tracers, which were, however, not determined in those coolants (mainly in ethanol) or which were optimized for different applications. Consequently, the two novel proposed dyes for the studied heat transfer liquids enabled a reliable temperature determination.

In summary, the presented measurements should enable the temperature determination in coolants as well as a simultaneous detection of the film thickness and film temperature in spray or film-cooling applications with an adequate illumination and a two-color camera system. The application of planar diagnostics for measurements in simplified heat transfer configurations and, finally, on real film cooling geometries is part of our future work.

## Figures and Tables

**Table 1:** Physical properties of the investigated heat transfer fluids [80,81,82,83,84].

Property	Unit	Fragoltherm F12	Marlotherm LH	Monoethylen–Glycol	Water
Density	g/cm^3^	7.630 (20 °C)	9.960 (20 °C)	1.110 (25 °C)	0.998 (20 °C)
Heat conductivity	W/(m∗K)	0.110 (20 °C)	0.132 (20 °C)	0.260	0.597 (20 °C)
Viscosity	mm^2^/s	1.60 (20 °C)	4.00 (20 °C)	16.31 (25 °C)	1.00 (20 °C)

**Table 2:** Coefficients of determination R^2^ for concentration-dependent absorption measurements.

Mixture	Coefficient of Determination R^2^
Water (Eosin-Y)	0.9994
WG20 (Eosin-Y)	0.9993
Glycol (Eosin-Y)	0.9988
Fragoltherm F12 (Nile red)	0.9990
Marlotherm LH (Nile red)	0.9992

**Table 3:** Coefficients of determination R^2^ for concentration-dependent emission measurements.

Mixture	Coefficient of Determination R^2^
Water (Eosin-Y)	0.9883
WG20 (Eosin-Y)	0.9563
Glycol (Eosin-Y)	0.9930
Fragoltherm F12 (Nile red)	0.9970
Marlotherm LH (Nile red	0.9923

**Table 4:** Filter selection for temperature-sensitive intensity ratio with selected filters containing corresponding stock numbers (SN, Edmund optics).

	Filter 1		Filter 2	
Solvent	CWL (nm)	SN	CWL (nm)	SN
Water	561	#12-152	540	#65-157
WG20	568	#65-160	540	#65-157
Glycol	580	#65-161	540	#65-157
Fragoltherm F12	560	#88-011	580	#65-161
Marlotherm LH	568	#65-221	632	#65-166

**Table 5:** Fitting parameters for the temperature two-color approach.

	p_1_	p_2_	p_3_	p_4_	R^2^	Valid
Water	1.27 × 10^−8^	−7.44 × 10^−6^	0.008919	−1.149	0.9996	(274 K–393 K)
WG20	−9.77 × 10^−8^	0.0001075	−0.02819	2.758	0.9999	(263 K–393 K)
Glycol	−3.77 × 10^−8^	3.57 × 10^−5^	0.001729	−1.103	0.9995	(253 K–393 K)
Fragoltherm F12	6.58 × 10^−9^	−1.44 × 10^−5^	0.01127	−1.033	0.9993	(253 K–393 K)
Marlotherm LH	−6.14 × 10^−7^	4.56 × 10^−4^	−0.06435	−1.999	0.9997	(253 K–393 K)

## Appendix A

The absorption of various heat transfer fluids is shown in Figure A1. All investigated heat transfer fluids except water (no absorption over the whole wavelength interval) showed significant absorption in the UV region studied.

The glycol concentration-dependent spectral shift of Eosin-Y (9.38 mg/L) in water/glycol is shown in Figure A2. The measurements revealed a linear shift of the peak towards longer wavelengths with an increasing glycol concentration.
